# Survivorship clinic attendance improves completion but not timeliness of cardiac surveillance post anthracyclines

**DOI:** 10.1186/s40959-025-00316-7

**Published:** 2025-02-25

**Authors:** Zac Forbes, Tegan Dunmall, Amanda Tey, Dominic Culligan, Pasquale L. Fedele

**Affiliations:** 1 University of Aberdeen, Aberdeen, Scotland; 2 Haematology Department, Monash Health, Clayton, VIC Australia; 3 Pharmacy Department, Monash Health, Clayton, VIC Australia; 4 Department of Haematology, Aberdeen Royal Infirmary, Foresterhill, Aberdeen, Scotland; 5 School of Clinical Sciences at Monash Health, Monash University, Clayton, VIC Australia

**Keywords:** Survivorship, Anthracycline induced cardiomyopathy, Anthracycline induced cardiotoxicity, Echocardiography, Echocardiogram, Cardiovascular, Heart failure, Anthracycline chemotherapy

## Abstract

**Background:**

Anthracycline induced cardiomyopathy (AIC) is an important complication of cancer management. Recent findings showed that with early identification and intervention, AIC may be fully or partially reversible. European society of cardiology (ESC) guidelines recommend a risk-stratified monitoring approach, including transthoracic echocardiogram (TTE) for all patients within 12 months post-treatment.

**Aim:**

Investigate the impact of a survivorship clinic on TTE follow up for AIC.

**Methods:**

Over a 5 year span, 235 patients with haematological malignancies received anthracycline chemotherapy ≥ 250mg/m^2^. The electronic medical records of these patients were reviewed. TTE outcomes were compared between survivorship and non-survivorship patients.

**Results:**

Survivorship patients received TTE in 88.6% of cases, whereas non-survivorship patients received TTE in 30.9% of cases. In survivorship patients, TTE was indicated for asymptomatic screening in 92.3% of cases. In non-survivorship patients the majority of TTE were for symptom investigation (78.0%). Chi-squared analysis found these results to be statistically significant (p value < 0.05).

**Discussion:**

Survivorship patients are nearly three times more likely to receive TTE monitoring for AIC. However, due to delayed clinic referral/attendance, only 36.4% received TTE within 1 year of treatment completion, in line with ESC guidelines.

**Conclusion:**

Survivorship clinics improve TTE monitoring for AIC, allowing early identification and potential intervention. However, reliance on this model alone may risk inadequate surveillance for patients who do not attend and delays in referral/attendance may impact monitoring timeliness.

## Background

Anthracycline induced cardiomyopathy (AIC) is a well described and potentially devastating complication of cancer treatment. The risk of AIC increases with cumulative anthracycline dose, with the European society of cardiology (ESC) guidelines highlighting that patients receiving ≥ 250mg/m^2^ doxorubicin equivalent are at high risk of AIC [1].

A recent prospective study found that in patients with AIC identified within a year of treatment completion and commenced on heart failure therapy, partial recovery was seen in 71% and total recovery in 11% [2]. This suggests that with early diagnosis and intervention, AIC may be reversible. To facilitate early diagnosis, ESC guidelines recommend risk stratification and cardiac monitoring for patients receiving anthracycline chemotherapy [1]. This includes class I/level B recommendation for a transthoracic echocardiogram (TTE) within 12 months of completing anthracycline treatment.

Survivorship clinics play an important role in the surveillance, prevention and management of treatment late effects. However, reliance on this model may risk inadequate surveillance for patients who do not attend and delays in referral or attendance may impact monitoring timeliness.

This single centre, retrospective study aimed to investigate whether survivorship clinic review improved timely requesting and completion of post-treatment TTE AIC surveillance in patients with haematological malignancies and high-risk anthracycline exposure.

## Methods

Patients administered anthracycline chemotherapy for haematological malignancy were identified from hospital pharmacy records as part of a quality assurance project, following ethics approval. Paediatric patients, patients with doxorubicin dose equivalent < 250mg/m^2^ and patients with incomplete treatment and/or follow-up information were excluded.

Data were collected from electronic medical records and included medical history and demographics, anthracycline dose, TTE details, survivorship clinic referral/attendance. The decision and timing of referral to the survivorship clinic is at the discretion of the treating haematologist, although the clinic encourages early referral post-treatment.

Data were analysed using IBM SPSS. A chi-squared test, Fischer’s exact test or students t test were used as appropriate. The Bonferroni method was used where appropriate to identify which values were statistically significant. Phi values indicated effect size (small: <0.3, medium 0.3–0.5, large: >0.5). Statistical significance was achieved at *p* < 0.05.

## Results

Anthracycline chemotherapy was administered to 571 patients with haematological malignancies over the five year period following 13th of December 2018. Following exclusions, 235 patients (59.1% male, median age: 60) received a cumulative doxorubicin-equivalent anthracycline dose ≥ 250mg/m^2^. Of these, 59 patients (25.1%) were referred to survivorship clinic, however only 44 patients (18.7%) had documented survivorship clinic review (‘survivorship group’), and 191 (81.3%) patients had no review (‘non-survivorship group’). The mean cumulative anthracycline dose was 297.1mg/m^2^ (SD: 18.2, range: 250–440) with no significant differences between groups. The survivorship group was significantly younger (survivorship: 44.5, non-survivorship: 62), with a significantly larger proportion of females (56.8%) and Hodgkin lymphoma patients (38.6%) (Table 1). The survivorship group also had significantly lower rates of pre-existing cardiac disease, hypertension, dyslipidaemia, T2DM and concomitant cyclophosphamide; but higher rates of mediastinal radiotherapy.

**Table 1:** TTE outcomes in survivorship and non-survivorship patients

Patient Characteristics				
Characteristic	Total sample *n* = 235	Survivorship group *n* = 44	Non-survivorship group *n* = 191	*p*-value
**Sex**				
Male, n (%)	139 (59.1%)	19 (43.2%)	120 (62.8%)	0.017
Female, n (%)	96 (40.9%)	25 (56.8%)	71 (37.2%)	0.017
**Age**				
Median (range)	60 (19–84)	44.5 (19–73)	62 (19–84)	< 0.001
**Dx**				
DLBCL, n (%)	117 (49.8%)	15 (34.1%)	102 (53.5%)	0.014
Hodgkin lymphoma, n (%)	44 (18.7%)	17 (38.6%)	27 (14.1%)	
Follicular lymphoma, n (%)	35 (14.9%)	5 (11.4%)	30 (15.7%)	
Anaplastic T cell lymphoma, n (%)	9 (4.3%)	1 (2.3%)	8 (4.2%)	
Marginal zone lymphoma, n (%)	5 (2.1%)	1 (2.3%)	4 (2.1%)	
Other*, n (%)	24 (10.2%)	5 (11.4%)	19 (9.9%)	
**Cumulative anthracycline dose (mg/m^2)**				
Mean (range)	297.1 (250–440)	298.3 (275–300)	296.9 (250–440)	0.42
**TTE Outcomes**				
**Outcome**	**All patients ***n* = **235**	**Survivorship group ***n* = **44**	**Non-survivorship group ***n* = **191**	**p-value**
Received TTE	41.7% (98/235)	88.6% (39/44)	30.9% (59/191)	< 0.001^a^
Received TTE within one year	22.6% (53/235)	36.4% (16/44)	19.4% (37/191)	0.018^a^
Received TTE for asymptomatic screening*	50.5% (49/97)	92.3% (36/39)	22.4% (13/59)	< 0.001^b^
Received TTE for symptom investigation*	51.5% (50/97)	10.3% (4/39)	79.3% (46/59)	< 0.001^b^

Baseline patient characteristics and rates of trans-thoracic echocardiogram performed in total, survivorship and non-survivorship groups. (TTE: Trans-thoracic echocardiogram, DLBCL: Diffuse large B cell lymphoma)

* 2 patients had multiple TTE performed for both symptomatic investigation and asymptomatic screening and were included under both categories

^a^ denotes a medium effect size (phi value)

^b^ denotes a large effect size (phi value)

In total 25.1% of patients were referred to the survivorship clinic, with 67.8% of referrals made within 1 year of treatment completion and 39.0% of patients attending their first appointment within a year of completion.

In the survivorship group, 88.6% underwent TTE, with 36.4% within a year of treatment completion. The indication for TTE was asymptomatic screening in 92.3% of survivorship patients. In the non-survivorship group, 30.9% of patients underwent TTE, with 19.4% within a year of treatment completion. The indication for TTE in non-survivorship patients was asymptomatic screening in 22.4% of patients. Differences between groups were significant for undergoing TTE, TTE within a year of treatment completion and TTE indication, with medium, medium and large effect sizes respectively.

Although our study was underpowered to compare TTE abnormalities between groups, 3.5% of total patients had symptomatic cardiac disease associated with anthracycline chemotherapy. A further 5.5% of total patients had an asymptomatic abnormality detected by post treatment TTE.

## Discussion

These findings demonstrate that survivorship clinic attendance significantly increases echocardiography surveillance for AIC. Patients attending the survivorship clinic were nearly three times more likely to undergo TTE. Importantly, in the majority of survivorship patients (92.3%), TTE was performed for asymptomatic screening. Conversely, TTE was only performed in a minority of patients not attending the survivorship clinic and concerningly when performed, was for investigation of cardiac symptoms in the majority (79.3%). While TTE surveillance was performed in 88.6% of survivorship patients, only 36.4% received TTE within a year of completing therapy. This means that even in the survivorship clinic, minimum ESC recommendations for cardiac surveillance are not being met. Late referral and attendance at the survivorship clinic are likely the primary reasons for late TTE follow up in patients referred to the clinic.

Our study was underpowered to contrast subsequent cardiovascular outcomes and AIC incidence. Previous studies would suggest that at this dose of anthracycline, 1.7% of patients will be affected by symptomatic AIC and 16.2% of patients will experience a decline in LVEF [3]. Our results are fairly comparable, with a slightly higher incidence of AIC (3.5%) and slightly lower incidence of asymptomatic LVEF decline (5.5%). Importantly, early recognition of this through TTE monitoring could increase the chance of reversal with intervention.

We found that younger patients were more likely to be referred for survivorship care. This drives the larger proportion of Hodgkin lymphoma and lower rates of co-existing cardiovascular disease and other risk factors seen in the survivorship group. While survivorship care in this younger population is important, older patients have higher rates of pre-existing cardiovascular disease and other AIC risk factors. This results in significant survivorship care needs which are not being adequately addressed.

The principles of survivorship care should be considered standard of care for all cancer patients. Our study showed that only 25.1% of patients at high risk of AIC were referred to the survivorship clinic, with even less referred within a year of treatment completion. This caused delayed post-therapy surveillance TTE beyond one year in close to two-thirds of survivorship patients. Given the restrictions on healthcare resourcing and increasing numbers of cancer survivors, a model of care relying on specialized survivorship clinic attendance is unfeasible [4]. Our findings suggest that survivorship clinics are also unlikely to facilitate timely AIC screening in the majority of patients.

AIC surveillance must therefore, be built into local institution chemotherapy protocols, however this alone is unlikely to ensure adequate and timely screening. Staff education, considering staff turnover and adequate resourcing are essential. Additionally, increasing use of electronic prescribing presents an opportunity to “automate” survivorship screening. For example, TTE assessment could be pre-programmed, and a request triggered based on cumulative anthracycline dose received. This could be extended to nurse-led survivorship screening clinics and/or multidisciplinary survivorship or cardio-oncology clinic referral.

## Conclusion

In this real-world study, survivorship clinic attendance significantly increased TTE monitoring for AIC. However, dependence on this resulted in inadequate cardiac surveillance for a large proportion of patients, particularly older patients who were less likely to be offered survivorship care. Furthermore, waiting for survivorship review delayed TTE in survivorship patients beyond ESC recommendations. Sustainable alternative strategies are required to optimise cardiac surveillance post anthracyclines.

## Data Availability

Full dataset can be anonymised and is available on request via email.

## Declarations

### Datasets

Please contact the authors for access to anonymized datasets.

## Abbreviations

AIC: Anthracycline induced cardiomyopathy
TTE: Trans-thoracic echocardiography
ESC: European society of cardiology
LVEF: Left ventricular ejection fraction
T2DM: Type 2 diabetes mellitus
IHD: Ischaemic heart disease
AF: Atrial fibrillation
CKD: Chronic kidney disease
MI: Myocardial infarction
